# Exploration of virtual body-representation in adolescence: the role of age and sex in avatar customization

**DOI:** 10.1186/s40064-016-2520-y

**Published:** 2016-06-16

**Authors:** Daniela Villani, Elena Gatti, Stefano Triberti, Emanuela Confalonieri, Giuseppe Riva

**Affiliations:** Psychology Department, Università Cattolica del Sacro Cuore, L.go Gemelli 1, Milan, Italy; CRIDEE, Psychology Department, Università Cattolica del Sacro Cuore, L.go Gemelli 1, Milan, Italy; Applied Tecnhology for Neuro-Psychology Lab, Istituto Auxologico Italiano, Via Magnasco 2, Milan, Italy

**Keywords:** Virtual self-representation, Avatar, Adolescence, Body image, Identity

## Abstract

The malleable nature of the self led researchers to investigate the meaning of virtual identity by exploring virtual self-representation through avatars and its association with users’ identity. The present study aims to investigate the changes in virtual body-representation in adolescence related to age levels and sex and the association with adolescents’ self-esteem and body esteem. Anthropometric features, body esteem and self-esteem were used to assess adolescents’ body image and identity. The scoring code of the “Drawing Me” graphical test was used to evaluate the avatars. The sample is composed of 63 adolescents of different ages—early, middle and late adolescence—balanced by sex. Results show that the creation of a digital avatar changes with age and is partially associated with adolescents’ perceptions in terms of body esteem and self-esteem. Moreover, the creation of avatars occurs differently for boys, who enrich their avatars with many sexual features, than for girls, who prefer to detail their avatars’ clothing to enrich them. Critical reflections and implications for psychological interventions that may use avatars to investigate adolescents’ identity in integration with other tools will be discussed.

## Background

Each individual has personal beliefs and selfconceptions—good self, bad self, not-me self, ideal self, possible self, ought self—that can be made accessible at a given moment (Markus 1977). Any of these selfconceptions can be activated at any particular time due to a host of factors that become salient in a social situation. The term “malleable self” (Markus and Kunda 1986) allows us to capture this dynamic, multidimensional conceptualization of identity (Oyserman 2004). Other theories (Butler 1990) challenge the concept of identity itself, implying that some aspects usually conceived as stable and predetermined features of a person, such as for example gender, are on the contrary constituted through action, discourse and behavior (“performative” identity).

In any case, the malleable nature of the self is germane to virtual self-representation in virtual environments (VEs), including Massively Multiplayer Online Role-Playing Games (e.g. World of Warcraft, Everquest) and Virtual Worlds (e.g., Second Life) where individuals can play with their identities. Within these VEs, users can transform and customize several attributes of their avatar, such as ethnicity, gender, body shape, face and clothes (Jin 2012). The possibilities for creating and customizing an avatar may range from a relatively basic icon—such as a two-dimensional character comprising pre-set elements chosen by the user (Vasalou et al. 2008), as in some social networking platforms (e.g., Yahoo Answers)—to a totally customizable avatar as found in three-dimensional virtual worlds (e.g., Second Life). The proliferation of virtual worlds, which have been described as laboratories for the construction of identities (Turkle 1997), has come hand in hand with an increase in attention toward these metamorphic avatars and the perception that one’s digital body is the desired rendition of one’s self (Comello 2009; Williams 2007; Yee and Bailenson 2007). The chance to experiment with various aspects of one’s personality and body image in VEs, by expressing or modifying various physical and psychological dimensions, has led researchers to investigate the meaning of virtual self-representation in order to better understand its association with users’ identity (Hoffner 2008; Williams et al. 2011). Also, if we consider the point of view of performative conception of identity, the virtual worlds can be seen as opportunities to enact performances that retroactively constitute one’s self conception. For instance, Cover (2012) has observed that social networking profiles (that include avatars) are tools to develop identity as a narrative in line with cultural demands.

The relationship existing between the creation and customization of a digital avatar and the identity of its user constitutes an open debate in the literature. On the one hand, some researchers recognize a substantial *discrepancy* between the individual’s actual self and his or her avatar (Trepte et al. 2009; Trepte and Reinecke 2010). When people migrate from the real world to an avatar-based virtual world, they may perceive a discrepancy between the actual self and the virtual self (i.e., virtual self-discrepancy) in virtual identity construction (Jin 2012; Ramirez and Wang 2008). Inspired by Higgins et al. (1985), ‘‘virtual self-discrepancy’’ can be conceptually defined as “the degree to which a user’s virtual identity represented in the form of an avatar in the virtual environment deviates from the user’s actual identity in the real world” (Jin 2012, p 2161). The construct of virtual self-discrepancy has been associated with users’ dissatisfaction with real life (Alsaker and Kroger 2006) and users’ intention to gain better mastery of the virtual world (Anderson et al. 2004). On the other hand, some researchers maintain that an avatar customized by an individual is substantially an extension of his or her self (Ata et al. 2011; Beasley and Standley 2002; Bell and Dittmar 2011), in that the avatar can be considered an expression of the individual’s actual identity (Bernasconi 2009; Bessiere et al. 2007). This coherence between individuals and their avatars have been found both related to personality, mental states, self-esteem and interests (Fong and Mar 2015; Kafai et al. 2010; Park and Henley 2007; Dunn and Guadagno 2012) and to behaviors exhibited within the virtual environment (Bessiere et al. 2007; McCreery et al. 2012). According to this perspective, avatars could express individuals “true selves” even if they live difficult situations or they are marginalized in the real world (Williams et al. 2011).

What emerges is that embodying an avatar is a recursive identity process and the fluidity of virtual self-representation encourages new interpretations of identity (Fox and Ahn 2013). This is particularly worthy of attention as virtual worlds become more popular among children and adolescents and appearance can play an important role in the ways avatars are used to communicate or express the self in a virtual world. Thus, those who are involved in the identity development process are called to show their bodies online, whether in a direct, indirect or virtual way. Furthermore, adolescents seem more likely than adults to identify themselves with their avatars and to develop emotional attachments toward them (Blinka 2008). As a consequence, there is growing attention about the relationship between the development of identity and virtual self-representation in adolescence (Klimmt et al. 2010; Milani et al. 2014).

We believe that investigating adolescents’ virtual self-representation represents an important challenge to take on for at least two reasons. First, avatar assessment allows to capture identity aspects in an unobtrusive way. Second, there is evidence that the ways in which users present themselves as avatars shape the behaviors of both users and perceivers (Nowak and Rauh 2005). This issue has important implications since users can portray themselves in a multitude of fashions. Self-presentations can affect online interactions between individuals in a manner that increases the risk of online sexual advances. Thus, monitoring avatar is important to protect young people, and especially girls, from risks related to excessive expression of their sexuality online and consequent online victimization (Noll et al. 2009).

In a recent study, Villani et al. (2012) found a promising method to analyze virtual body representation and compared it with body drawing. Results indicated that the adolescents put more sexual body, facial and clothing features in their avatars than in their drawings. More, girls included more sexual characteristics than boys, such as breast or hips and make up details. Although authors found that through the avatar it is possible to investigate important aspects of body representation, it is critical to consider that body representation changes through adolescence (Verstuyf et al. 2014). Recent studies (Confalonieri 2011; Gatti et al. 2014) show that there is a relationship among physical changes through puberty, psychological development and body representation; adolescents who are satisfied with their own bodies and faces represent themselves with a greater number of secondary sexual characteristics, many body details and coherent body shape and proportions. For this reason, a multidimensional analysis of avatar aimed at exploring differences among age phases and its association with adolescents’ body image and identity is needed.

Many studies agree that the most important individual factors influencing sense of identity in adolescence are age and sex (Holmqvist and Frisen 2012; Weichold and Fasche 2010; Ata et al. 2011; Gatti et al. 2014). Regarding age, body representation and body esteem change at different ages (early, middle and late adolescence) (Verstuyf et al. 2014) according to adolescents’ physical development (puberty). It seems that early adolescents are more satisfied with both their physical appearance and their weight and have higher levels of self-esteem than middle adolescents (Ciuluvica et al. 2010). Literature about late adolescence shows conflicting results: in case of coherence between adolescents’ body image and self-perception, adolescents increase satisfaction with their bodies; otherwise, adolescents could show internalized or externalized problems (Weichold and Fasche 2010). The research has shown that also avatar creation is sensitive to age differences. For example, Martey et al. (2013) found that appearance of participant avatars is a significant predictor of their age. Specifically, old users tend to customize avatar with more traditional appearance, such as representing themselves as something they perceived as accurate to their offline appearance (Reed and Fitzpatrick 2008). Younger players, that are players under thirty-5 years-old, are more likely to experiment their appearances. Nevertheless, these studies have been focused on adult age and, to our knowledge, there is still no research investigating changes in avatar customization depending on different phases of adolescence. For this reason, also this aspect constitutes an interesting aim to analyze in the present study.

Regarding sex, females appear more worried about their body appearance and more dissatisfied with their weight and they report lower self-esteem than males (Shea and Pritchard 2007; Bell and Dittmar 2011). This difference between girls and boys could be emphasized by the centrality of appearance in the female gender role, typical of western cultures. Avatars have been already considered an interesting field of research for what regards the representation of sex differences. For example, as shown by Beasley and Standley (2002), there is considerable gender role stereotyping in video games that still promotes unequal representations of gender characteristics, with male characters over-represented than female ones, and female characters often represented with unrealistic bodies and sexually-revealing clothing. Actually, this aspect is only indirectly related to the topic discussed here, in that research on gender role stereotyping in video games does not relate to avatars only, but also to non-playable characters. However, it may be important to consider that some adolescents can be influenced in their customization preferences by media-conveyed stereotypes. Indeed, adolescents are characterized by an intense exploration of all the components necessary for identity development (Burgess et al. 2007); these components include vocational and interpersonal roles. In a recursive way, literature highlights a strong link between exposure to gender roles depicted in the media, including videogames, and the attitudes towards gender roles in the everyday life (Kolbe and LaVoie 1982; Anderson et al. 2004). Therefore, analyzing whether there are differences in how male and female adolescents virtually-represent themselves represents a critical goal even for future studies aimed to understand what specific gender behaviors users may learn from *playing* with their avatars.

Starting from these premises the main goal of this study is to explore changes in virtual self-representation in adolescence. Specifically, we are interested in verifying these two principal hypotheses:

### H1

There are differences between adolescents’ virtual representations (avatars) according to their age levels and sex. In particular, according to the literature, we expect that (1) late adolescents will include a higher number of secondary sexual characteristics when creating their avatars than will early adolescents and that (2) adolescent females will create avatars with more sexual details compared to adolescent males.

### H2

Adolescents’ virtual representations are associated with their identity (body image and self-esteem). Specifically, we expect that adolescents characterized by low self-esteem and low body satisfaction will create avatars with less detail and poor sexual characterization.

Furthermore, we wondered if adolescents perceived a discrepancy between the actual self and the virtual self in avatar construction (RQ1).

In order to address these hypotheses and the last research question, we adopted a research approach involving early, medium and late adolescents who created and customized avatars to represent themselves. Avatars have been codified in their features basing on the “Drawing Me” method, that has been already used in this field (Villani et al. 2012). Moreover, the participants provided anthropometric measures, self-esteem, body-esteem data, and their opinion on self-avatar discrepancy filling in validated or ad-hoc questionnaires.

## Methods

### Participants

The sample included 63 adolescents between 11 and 19 years of age (M = 14.70; SD = 2.5) who completed all study measures. Following previous literature stating that body representation and body esteem change at different ages (early, middle and late adolescence) according to adolescents’ physical development (Blos 1967; Finlay et al. 2002; McCabe and Ricciardelli 2005; Gatti et al. 2014; Verstuyf et al. 2014), the sample included three age levels: 26 early adolescents aged 11–13 (M = 11.92; SD = .2), 15 middle adolescents aged 14–16 (M = 15.33; SD = .5) and 22 late adolescents aged 17–19 (M = 17.55; SD = .7). Participants were also balanced by sex (males = 29; females = 34).

Even though age and gender are the two principal factors in adolescents’ body perceptions investigated in this study, other individual variables, such as the body mass index (BMI), could also influence one’s perception and body representation (Jones and Crawford 2005; Fenton et al. 2010). Thus, we selected adolescents with normal range of BMI to avoid pathologies that could affect their body image and, consequently, their avatar creation.

Early adolescents were recruited in a secondary school, while middle and late adolescents were recruited in a high school, from the metropolitan area of Milan, Northern Italy. Participants came from different classes within these two schools. They had Italian backgrounds and came from upper-middle socioeconomic classes.

Ethical approval was gained through the University research ethics committee, which required informed consent from each participant’s parents.

### Procedure and measures

Psychological assessment procedures and data collection of anthropometric clinical features (weight, height, and BMI) were performed by the researchers.

The administration of instruments took two sessions: during a class period, the adolescents completed questionnaires (first session); then, in a small group setting, they created their own avatars (second session). Finally, the participants answered two items about avatar self-discrepancy. Researchers monitored all processes and ensured that the students completed the questionnaires by themselves and in a silent and private context. Each student was free to drop out of the research any time and to express to the researchers any doubts or uncomfortable feelings. Details about the instruments we used are reported below.

Regarding adolescents’ body image and identity, we assessed:

1. *Anthropometric features* BMI was used to estimate a healthy body weight based on a person’s height. It is the most widely used diagnostic index to identify weight problems, usually to estimate whether individuals are underweight, overweight, or obese. The formula universally used is kg/m^2^. We just checked the BMI distribution to exclude outliers.
2. *Body esteem* The Body Esteem Scale (BES, Mendelson et al. 2001; Italian version by Confalonieri et al. 2008) consists of 14 items, and respondents indicate their degrees of agreement on a five-point Likert scale ranging from 0 (never) to 4 (always). Seven negative items are reverse-scored. The scale measures three factors: *attribution* (the evaluation attributed to others about one’s body and appearance), *weight* (satisfaction with one’s weight), and *appearance* (general feeling about one’s appearance). For these data, the three subscales had an adequate reliability (attribution: alpha = .68; weight: alpha = .84; appearance: alpha = .76).
3. *Self*-*esteem* The Rosenberg Self-Esteem Scale (Rosenberg 1965; Italian version by Prezza et al. 1997) is the most widely used measure of global self-esteem and has been determined to be valid and reliable among students (Rosenberg 1989). Responses to the 10 items are rated on a four-point Likert scale (strongly disagree to strongly agree), yielding scores between 10 and 40. The scale measures three factors: *self*-*esteem* (global self-worth), *self*-*deprecation* (criticism about self) and *self*-*respect* (praise of self), showing a high internal consistency (Cronbach’s alpha = .84) and a good test–retest correlation (r = .76).
  Regarding avatar design, different studies showed that avatar creation/customization is strongly influenced by the cultural aspects of the virtual world in which the avatar is expected to be used (Ducheneaut et al. 2009; Triberti and Argenton 2013). To control this aspect and to avoid differences related to virtual contexts in which avatars would be used (e.g. a videogame or a social network), we decided to use appropriate software to allow participants to create their own avatars. According to the instruction of the “Drawing Me” Test (for details see the *avatar scoring* paragraph) adolescents were instructed as follows: “Please try to depict yourself by drawing the way you would want to present yourself to a person who was interested in you but did not know you. You are free to represent yourself in the most appropriate way, trying to communicate to the other person who you are and what your characteristics are”.
4. *Avatar creation* Avatars were created by the participants using the free online software Meez (www.meez.com), which allows the user to choose several aspects related to the prototypic figure, such as gender, as well as more specific details, such as height, body shape, clothes and environment.
5. *Avatar scoring* To evaluate the virtual avatar, we used the scoring code of the “Drawing Me” graphical test. The Drawing Me test, originally created for assessing body image perception of adults (Witkin et al. 1962) and then adapted in Italian and rearranged for adolescents (Confalonieri 2011) aims at identifying, through a graphic representation what adolescents think about their body in terms of perceptions, attributions, satisfaction, or self-perceived integration of body districts. The use of this test is consistent with a good deal of research literature that suggests the utility of incorporating drawings into assessment practices. The drawing method, in fact, offers the opportunity to obtain qualitative information that may not be easily retrieved through conventional paper-and-pencil verbal tests and, as such, may broaden the range of available information (Matto 2006). Concerning avatar, the efficacy of the Drawing Me Test in analyzing the virtual body representation has been already tested (Villani et al. 2012). Although we recognize that other avatar coding schemes exist, we consider it as the most appropriate to investigate virtual body representation of adolescents (Confalonieri 2011; Gatti et al. 2014; Villani et al. 2012).
  Specifically, for avatar scoring we considered two scales: level of detail and level of sexual characterization. Each scale is structured in three different subscales scored from 1 to 3, assessing the *quantity* of specific characteristics. In other words, the focus is not on the characteristics resembling or not some arbitrary quality standard (e.g., beard of one type or another; heavy or light make up; etc.), but on how many of sexual characteristics are actually present in the avatars (see Table 1).
  After the training period, three blinded independent examiners rated every avatar. The accordance between judges, measured by Cohen’s K coefficient, was .84.
6. *Avatar Self*-*Discrepancy* As a final measure, two items on a five-point Likert scale measured the avatar self-discrepancy (“How similar is your avatar to how you would like to be?”) and the avatar self-coherence (“How much is your avatar like you?”).

**Table 1:** Avatar grid code

Scale	Subscales	Subscale description	Evaluation
Detail Level	Body details	Numbers of body parts (arms, legs, face, feet, hands, etc.)	1 = one or more body parts are missing 2 = all parts are represented but one or more are hidden 3 = all body parts are presented and visible
	Face details	Numbers of facial features (eyes, nose, lips, etc.)	1 = one or more facial features are missing 2 = all features are represented but one or more are hidden 3 = all features are presented and visible
	Clothing details	Numbers of garments and accessories (tops, pants, skirts, hats, etc.)	1 = one or more clothing items are missing 2 = all clothing items are represented but one or more are hidden 3 = all clothing items are represented
Sexual Characterization Level	Body sexual characterization (body features driven by physical development)	Number of sexual features (breasts or hips for girls; body hair or muscles for boys)	1 = no sexual body elements 2 = one sexual body element is represented 3 = two or more sexual body elements are represented
	Facial sexual characterization (facial features driven both by physical development and socio-cultural aspects)	Numbers of facial sexual features (makeup or eyelashes for girls; beard or square jaw for boys)	1 = no sexual facial elements 2 = one sexual element is represented 3 = two or more sexual facial elements are represented
	Clothing sexual characterization (gendered nature of the clothes)	Numbers of gendered clothing items (skirts and tops for girls; pants and sweatshirts for boys)	1 = clothes don’t belong a specific gender 2 = one clothing item identifies the gender

## Results

Before analyzing adolescents’ differences in avatar creation and the association with their identity, we checked the BMI distribution to eventually exclude participants with weight problems but we did not find outliers. BMI increased through adolescence and revealed a normal growth of the sample (Bernasconi 2009).

Descriptive data from body esteem, self-esteem and BMI by age and sex are reported in Table 2.

**Table 2:** Descriptive data from BES and RSE questionnaires by age and sex (M and SD)

	Early adolescents		Middle adolescents		Late adolescents	
	Female	Males	Females	Males	Females	Males
Appearance (BES)	8.71 (2.26)	6.44 (2.18)	7.85 (2.27)	6.62 (3.58)	8.10 (2.68)	8.25 (2.92)
Attribution (BES)	7.82 (3.13)	5.89 (1.83)	7.00 (3.16)	7.75 (3.15)	11.70 (2.16)	8.75 (2.41)
Weight (BES)	7.94 (1.67)	7.00 (1.32)	6.00 (2.16)	6.87 (1.35)	7.10 (1.37)	6.83 (1.58)
Self-deprecation (RSE)	2.41 (.35)	2.32 (.45)	2.32 (.46)	2.19 (.33)	2.20 (.46)	2.29 (.30)
Self-respect (RSE)	3.39 (.42)	3.44 (.33)	3.28 (.55)	3.58 (.38)	3.27 (.71)	2.25 (.38)
Self-esteem (RSE)	2.71 (.23)	2.65 (.30)	2.61 (.36)	2.61 (.17)	2.52 (.31)	2.58 (.20)
BMI	17.37 (1.97)	16.31 (1.36)	19.41 (2.15)	17.53 (2.00)	20.38 (3.02)	19.36 (2.06)

### Differences between adolescents’ virtual representations (avatars) according to their sex and age

Since the scoring of avatar coding was carried out on an ordinal scale, it was not possible to use parametric statistics. Thus, to examine statistically the relationship between age, sex and adolescents’ virtual representations, firstly we tested a series of log-linear models. As the interaction effects between the three variables were not significant, we used not parametric tests to analyze the effects of age (Kruskal–Wallis test) and sex (Mann–Whitney test) on avatar coding (descriptive data are presented in Table 3).

**Table 3:** Descriptive data of avatar code by age and sex (M and SD)

	Early adolescents		Middle adolescents		Late adolescents	
	Female	Males	Females	Males	Females	Males
Body details	2.52 (.51)	2.37 (5.17)	2.71 (.75)	2.87 (.35)	2.70 (.48)	2.83 (.38)
Face details	2.71 (.47)	2.12 (.35)	2.86 (.37)	2.87 (.37)	2.80 (.42)	3.00 (.01)
Clothes details	2.17 (.53)	2.25 (.46)	2.43 (.53)	1.87 (.64)	2.10 (.31)	1.67 (.49)
Total details	7.41 (.94)	6.75 (.71)	7.14 (2.11)	7.50 (.75)	7.60 (.69)	7.58 (.51)
Body sexual char.	2.06 (.66)	2.00 (.01)	2.71 (2.43)	2.62 (.51)	2.40 (.51)	2.58 (.51)
Facial sexual char.	2.12 (.60)	2.77 (.46)	2.28 (2.42)	3.00 (.01)	2.40 (.51)	3.00 (.01)
Clothing sexual char.	2.59 (.72)	2.75 (.46)	2.28 (.75)	2.00 (.92)	2.70 (.48)	2.25 (.96)
Total sexual char.	6.67 (1.52)	7.50 (.75)	6.00 (1.73)	7.62 (1.30)	7.60 (1.26)	7.83 (1.27)

Concerning age differences, the scores of details related to facial features (χ2 = 10.685, df = 2, p < .01, η2 = .175) significantly changed. Specifically, late (U = 168.000, p < .005, η2 = .174) and middle (U = 122.500, p < .05, η2 = .124) adolescents gained higher scores than early adolescents and represented themselves with more facial details (e.g., they include eyelashes, eyebrows, or lips). No other significant differences were found related to body (χ2 = 4.713, df = 2, p = .095) and clothing (χ2 = 4.904, df = 2, p = .086) details.

Furthermore, we found significant differences related to body sexual characterization (χ2 = 6.950, df = 2, p < .05, η2 = .116) and face sexual characterization (χ2 = 6.675, df = 2, p < .05, η2 = .109). Also in this case, the sexual characterization both of body and facial features increased with the age of adolescents: late adolescents represented themselves with more sexual features (U = 179.000, p < .05, η2 = .110) (e.g., facial hair for boys and makeup for girls) and with more secondary sexual characteristics that indicated their gender (U = 165.000, p < .05, η2 = .159) than early adolescents. No significant differences were found related to clothing sexual characterization (χ2 = 4.000, df = 2, p = .118).

**Fig. 1:**
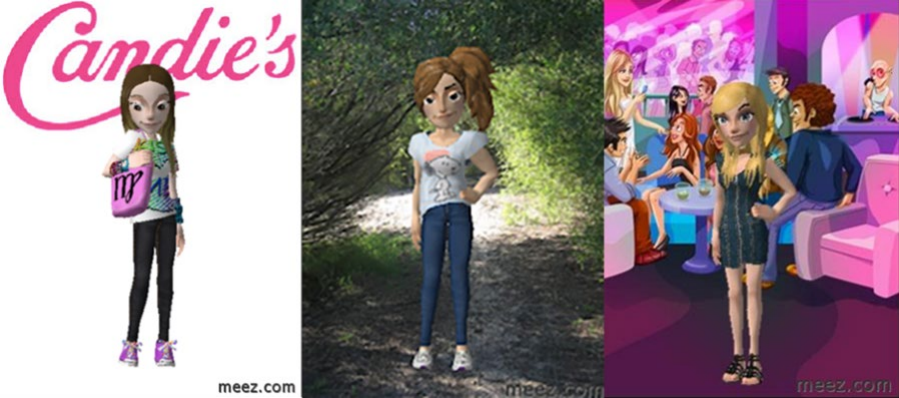
Early, middle and late female adolescents’ avatars

**Fig. 2:**
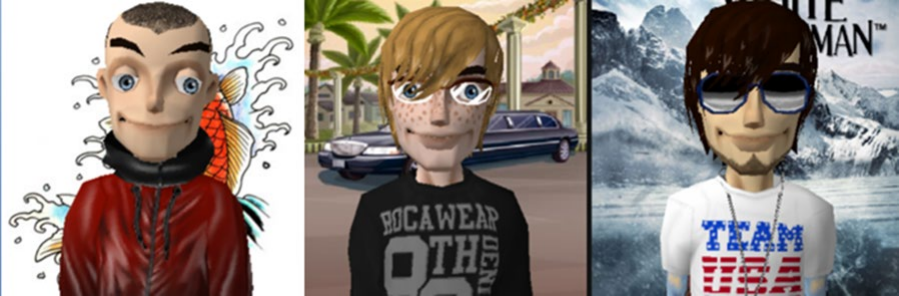
Early, middle and late male adolescents’ avatars

Below are examples of adolescents’ avatars according to their age level (Fig. 1 shows female changes and Fig. 2 shows male changes).

Concerning sex, we found significant differences in clothing details (U = 349.500, p < .05, η2 = .082), but no differences in facial (U = 452.000, p = .654) and body (U = 398.000, p = .265) details. Furthermore, facial sexual characterization (U = 185.000, p < .01, η2 = .373) and sexual total scale (U = 330.500, p < .05, η2 = .073) changed between male and females. Specifically, females tended to create avatars with much more clothing and ornamentation than males, whereas the males added more sexual features on their face, body and clothes than females.

**Fig. 3:**
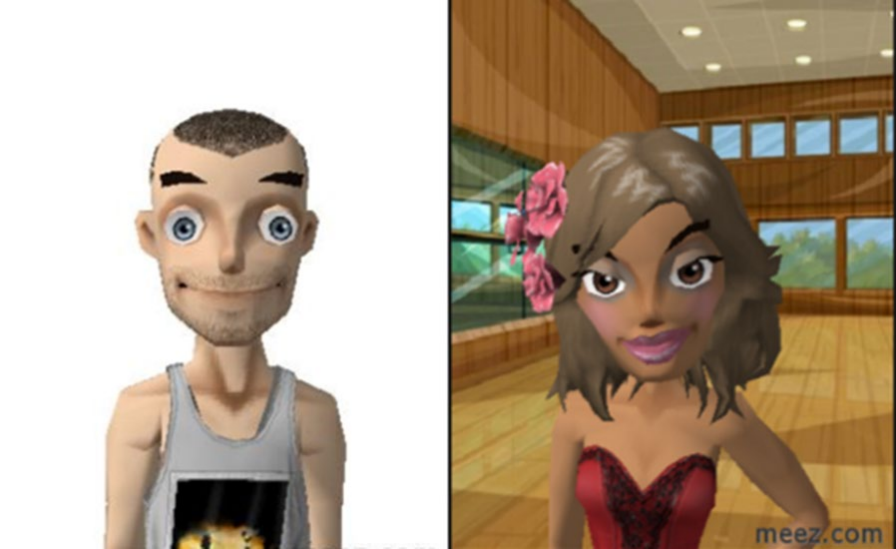
Male and female adolescents’ avatars

Figure 3 shows a female’s avatar wearing flower in the hair and male’s avatar with beard.

### Relationship between adolescents’ virtual representations and their body image and self-esteem

We conducted Spearman’s Rho correlations between the self-report measures (BES, RSE) and virtual representation (avatar coding) including all participants. The matrix of correlations is represented in Table 4.

**Table 4:** Bivariate correlations with exact Spearman Rho and p values between BES, RSE subscales and avatar scoring

	Appearance	Attribution	Weight	Self-deprecation	Self-respect	Self-esteem	Body details	Face details	Cloth. details	Total details	Body sexual char.	Facial sexual char.	Cloth. sexual char.	Total sexual char.
Appearance	–	−.010, p = .936	229, p = .071	229, p = .071	−.268,*p = .034	.131, p = .307	.309,*p = .015	.060, p = .343	.115, p = .372	.326,**p = .010	−.020, p = .877	−.080, p = .537	−.039, p = .764	−.094, p = .466
Attribution	–	–	.041, p = .751	−.346,**p = .005	.169, p = .186	−.268,*p = .027	.175, p = .176	.101, p = .433	−.102, p = .430	.159, p = .218	−024, p = .854	−.011, p = .930	.179, p = .163	.110, p = .193
Weight	–	–	–	.109, p = .397	−.105, p = .411	.094, p = .466	.035, p = .789	−.117, p = .366	−.033, p = .797	−.048, p = .714	−.047, p = .719	−.122, p = .344	−.008, p = .949	−.086, p = .508
Self-deprecation	–	–	–	–	−.305*, p = .015	.872**, p = .000	.104, p = .424	.031, p = .811	−.044, p = .733	.119, p = 358	−.034, p = .793	−.240, p = .061	.051, p = .692	−.084, p = .514
Self-respect	–	–	–	–	–	.133, p = .198	.007, p = .958	−.034, p = .790	.170, p = .186	.169, p = .189	.153, p = .239	.262*, p = .040	.155, p = .230	.268*, p = .035
Self-esteem	–	–	–	–	–	–	.108, p = .405	−.010, p = .936	.064, p = .623	.209, p = .103	.020, p = .881	−.123, p = .340	.137, p = .288	.040, p = .759
Body details	–	–	–	–	–	–	–	.173, p = .182	−.160, p = .219	.520**, p = .000	.362**, p = .004	.113, p = .386	.082, p = .530	.237, p = .066
Face details	–	–	–	–	–	–	–	–	−.059, p = .646	.569**, p = .000	.289*, p = .024	.186, p = .147	−.242, p = .058	.045, p = .726
Cloth. details	–	–	–	–	–	–	–	–	–	.501**, p = .000	.034, p = .797	−.046, p = .724	.240, p = .060	.115, p = .373
Total details	–	–	–	–	–	–	–	–	–	–	.395**, p = .002	.161, p = .212	.138, p = .284	.308*, p = .015
Body sexual char.	–	–	–	–	–	–	–	–	–	–	–	.380**, p = .003	.221, p = .088	.743**, p = .000
Facial sexual char.	–	–	–	–	–	–	–	–	–	–	–	–	−.030, p = .817	.575**, p = .000
Cloth. sexual char.	–	–	–	–	–	–	–	–	–	–	–	–	–	.665**, p = .000
Total sexual char.	–	–	–	–	–	–	–	–	–	–	–	–	–	–

* p < .05

** p < .01

Body esteem and self-esteem appear to be partially related to avatar creation. Specifically, the appearance (BES subscale) is positively correlated with avatar body details and total details. Thus, adolescents with a good general feeling about their appearance represent themselves by including all body parts visible, a large number of facial features and of garments and accessories. The evaluation attributed to others about one’ body and appearance (attribution) and the satisfaction with one’s weight (weight) are not associated with avatar creation.

Furthermore, the self-respect (RSE subscale) is positively correlated with avatar facial sexual and total sexual characteristics. Thus, adolescents that pride themselves created avatar with a great number of secondary sexual features. The global self-worth (self-esteem) and the criticism about self (self-deprecation) are not associated with avatar creation.

### Adolescents’ virtual-self discrepancy

We conducted a repeated measure ANOVA to compare the answers to avatar self-coherence (“How much is your avatar like you?”) with the answers to self-avatar discrepancy (“How similar is your avatar to who you would like to be?”). We found no significant differences between them (F_(1,54)_ = 1106; p = .298): the participants answered that the avatars were equally similar to both their own real selves and their ideal selves.

Furthermore, we performed a correlation analysis between the avatar-self coherence and avatar-self discrepancy questions, finding a significant positive relation between the two: ρ = .454, p = .001. It appears that no avatar-self discrepancy can be found among the sample, as the participants evaluated their own avatars as equally similar to their own real selves and their ideal selves.

Finally, we performed a correlation analysis between the avatar-self discrepancy and self-esteem, but we did not find significant correlations (self-deprecation: ρ = .168, p = .219; self-respect: ρ = .129, p = .347; self-esteem: ρ = .244, p = .072).

## Discussion

The present research aims to test the effectiveness of a multidimensional assessment of avatar in adolescence. To reach this goal we combined quantitative and qualitative measures to obtain a whole picture of virtual body image representation in adolescence. Adolescence is a period of life characterized by many important physical and psychological changes that may influence the identity development (Harter 2003; Alsaker and Flammer 2006; Smolak and Stein 2010; Fenton et al. 2010; Confalonieri and Gatti 2011). Since many studies have shown that the most important individual factors influencing the sense of identity in adolescence are age and sex (Holmqvist and Frisen 2012; Weichold and Fasche 2010; Ata et al. 2011; Gatti et al. 2014), and these factors are related also to avatar appearance in adult samples (Martey et al. 2013; Reed and Fitzpatrick 2008; Beasley and Standley 2002), we first investigated whether these factors influence the adolescent virtual self-representation.

Our first hypothesis has been confirmed. Results show that the creation of a digital avatar changes according to age level. Specifically, the more adolescents grow, the more they show, enriching the virtual representations of themselves with facial details and with facial and body sexual features. The results confirm that, during the transition from childhood to adulthood, the mental representation of one’s own body becomes more precise and accurate, including more details and sexual features. Probably, adolescents accurately depict changes in their real world bodies or personal style that also occurred with age, as previously showed in adult population (Reed and Fitzpatrick 2008). The detailed representation of secondary sexual features indicates that the body becomes more “personalized”. This “personalized” body is very important for reaching a body identity that will be integrated into a global identity at the end of adolescence (Alsaker and Kroger 2006; Bucchianeri et al. 2013).

Moreover, the creation of avatars occurs differently for boys than for girls. Males enrich their avatars with many sexual features (face, body and clothes) that underline their sex, whereas females prefer to detail their avatars’ clothes and enrich them with jewelry and objects. Concerning the gendered nature of the clothes we have to consider that the free online software used is probably influenced by socio-cultural aspects that have restricted the choice opportunities. Perhaps we found differences only in clothing details for this reason. Thus, clothing might be one opportunity for young women to explore and publicly present their sexuality (Gleeson and Frith 2004). Indeed, this permits to reconsider the contribution of performative identity theories (Butler 1990; Cover 2012; David et al. 2006): both the acts of males adding sexual body features and those of females adding jewelry and objects constitute identity performances, that are, repeated acts devoted to the construction of identity. In other words, avatar details and sexual characterization can be seen as a way in which an adolescent’s gender become intelligible through particular actions in the virtual world, as well as “girling” allow adolescents become identified and recognized as girls in the real world (Butler 2004; Nayak and Kehily 2006). This aspect should be carefully considered by future studies focusing on self-presentation online. In fact, previous research has found that there are some risks related to female hyper-sexualized representation online. Adolescent girls’ tendency to create sexually provocative avatars to represent themselves was tied to Internet victimization (Noll et al. 2009), in that girls who prefer provocative avatars (in terms of body and clothes) have been found more likely to receive online sexual advances.

As we considered avatar as the virtual representation of the adolescents’ actual identity, we would like to verify also the association between virtual self-representation and their body esteem and self-esteem. Our second hypothesis has been partially confirmed. We found some associations between the characteristics of the avatars and adolescents’ perceptions in terms of body esteem and self-esteem. It emerged that those with a positive feeling about their appearance tended to add more facial, body and clothes details to their own avatars. The satisfaction with one’s weight and the evaluation attributed to others about one’ body and appearance are not associated with avatar creation, which features range from limited list of options. Probably, the drawing method allows obtaining more information related to adolescents body esteem than avatar creation. Moreover, those with more self-respect created avatar with a great number of secondary sexual features. These correlations reinforce the link between physical and psychological development and highlight the important role of body changes in influencing body esteem, self-perception and self-worth (Alsaker and Kroger 2006).

Regarding our research question, we investigated whether the avatars created by the adolescents could be considered subjective representations of how they think they actually are (“real” selves”) real selves or how they desire to be in their imagination or in the future (“ideal selves”). To answer to this question, we analyzed avatar-self coherence versus avatar-self discrepancy in the context of our sample. Surprisingly, the results showed that the adolescents considered their avatars to be equally similar to both their real and ideal selves. In this sense, the adolescents of our sample seem to be characterized by an advanced level of self-construction, since their ideal selves appear not dissimilar from their real ones, if not substantially overlapping. This interpretation is confirmed by the significant positive correlation between the avatar-self coherence and avatar-self discrepancy questions.

To better understand this result, which is part of the debate on the relationship between the creation and customization of a digital avatar and the identity of its user, we have to consider two critical factors. First, adolescents characterized by high levels of self-esteem and a coherent body image constituted our sample. Thus far, avatar-self discrepancy has been primarily investigated in adults. A few studies have focused on adolescents, but their participants were characterized by specific pathologies, like online gaming addiction (Wan and Chiou 2006; Smahel et al. 2008) or a poor level of psychological well-being (Bessiere et al. 2007). Self-discrepancy theory suggests that psychological well-being is closely related to a person’s actual self (“me as I am”) versus his or her ideal self (“me as I would like to be”). People with larger real–ideal self-discrepancies thus have higher depression and lower self-esteem. Moretti and Higgins (1990) found the actual—ideal self-discrepancy to predict significant variance in self-esteem but this effect was only found when an idiographic, rather than a nomothetic, self-discrepancy measure was used. Even if we did not find significant correlations between avatar-self discrepancy and self-esteem, we can not exclude an effect due to the measure we have used.

Second, we have to consider that the adolescents in our sample created their avatars without a context of use. Different virtual worlds are characterized by the support they offer to specific activities that can bring out specific aspects of the user’s identity (McCreery et al. 2012). Moreover, the creation/customization of avatars by videogame players is strongly influenced by the cultural aspects of the virtual world in which the avatar is expected to be used (Ducheneaut et al. 2009; Triberti and Argenton 2013). Last but not least, users’ personal preferences and communicative intentions may influence natural avatar creation; for example, one may prefer a cartoon-like design trying to be funny, or a monstrous appearance in order to intimidate others (Triberti and Argenton 2013). For this reason, we used an avatar creation/customization program in order to invite participants to simply represent themselves through avatars, without the intention of later guiding them into an online videogame or a social networking platform. By doing so, we ensured that the avatar creation by the participants was related to self-expression only, and not to the possible intentions of a player who expects to enter a virtual world containing social relationships and cultural features. We think that if a user is asked to digitally re-create his or her identity *with the purpose* of communicating with others, he or she could be driven to modify it according to a desired rendition of the self. In contrast, according to our research, when an avatar-creation program is used to create an avatar that is not expected to be used elsewhere, the result can be considered more as an indication of how the *real* self of the user is constructed and perceived. It’s important to consider that the experimental manipulation allowed to focus on the specific phenomenon of virtual self representation but could have reduced the overall ecological validity of the study. Thus, this result may be considered with caution as it is not representative of any natural situation in which avatars are created by computer users.

## Conclusions

To conclude, by extending previous results of Villani et al. (2012), which explored body representation in adolescence through the use of avatars, this study shows that, in a balanced sample characterized by high levels of self-esteem and a coherent body image, avatar creation becomes more detailed as adolescents’ age levels increase. More, virtual body representation is partially associated with body image and self-esteem. Data are promising and confirm the importance of analyzing avatar in a multidimensional way and integrating both quantitative and qualitative measures to obtain a whole picture of virtual body image representation in adolescence. Specifically, future qualitative measures are encouraged to use scales name coherent with the content investigated and to identify continuous variables in order to allow powerful statistical analyses. Given the proliferation of virtual worlds and social networks among adolescents, the present research might offer a particularly promising window into the comprehension of the psychological correlates of virtual self-representation of adolescents, in terms of what they express and say about themselves through the customization of digital figures. Other future challenges for research regard the elaboration of proper tools and/or experimental settings to investigate the other factors that may mediate the relationship between self representation and avatar creation, such as personal preferences/intentions, cultural characteristics of the virtual world, and type of avatar among others. For instance, literature (Sanford et al. 2015; Schwind et al. 2015) argues that people may not like avatars with a cartoon-like design, and this could be systematically related to age, with older subjects preferring realistic virtual figures.

On the one hand, implications of the present study for the “natural context” of virtual worlds and gaming should be considered with caution: indeed, in the present study avatar creation was manipulated with specific guidelines for the participants, in order to focus their customization effort on self-representation. Nevertheless, we should consider that adolescents who create/customize avatars to interact or play online could not have intention of represent themselves. Furthermore, the use of a coding scheme based on avatars’ features quantity could represent a limitation of the study. Although this method has already been used in the literature with satisfying results (Villani et al. 2012), it may be considered controversial, and the recourse to more objective and repeatable coding methods would be preferable for future studies.

On the other hand, the present study offers implications for both psychologists and educators who would make use of avatar creation/customization independently of the virtual worlds context in which they are typically expected to be used. In order to generate preventive measures to help adolescents through their developmental task, professionals can support adolescents to reflect upon their own self-perception in using avatar creation/customization. In conjunction with other proper instruments, the use of avatars can be a resource to promote adolescents’ self-knowledge, body esteem and self-esteem and, at the same time, to help them in identifying the possible influences of gender stereotypes along with the risks of internet victimization. Reinforcing skills such as self-esteem and the abilities to withstand social comparisons with peers and to accept the outcomes of bodily change could help adolescents to face the process of their pubertal development with better preparation and a greater ability to reach a satisfactory image of their own adult body. These processes could be enhanced by avatar designers careful in proposing avatar features that promotes online victimization prevention. Future researches are encouraged to investigate virtual body representation through avatar in adolescence with longitudinal studies, and to consider also specific clinical sample to understand if the avatar creation could be usefully integrated within therapeutic or educational treatments.
